# *Cistanche deserticola*: A Homology-of-Medicine-and-Food Herb with Intestinal-Moistening and Laxative Effects

**DOI:** 10.3390/nu18213645

**Published:** 2026-11-01

**Authors:** Zhen-Hong Cheng, Ning Liu, Yue Liu, Lin Ma, Xiao-Bing Lan, Chun-Hao Zhu, Chao-Kun Wei, Jian-Qiang Yu, Wei Wei

**Affiliations:** 1 College of Pharmacy, Ningxia Medical University, 1160 Shengli Street, Yinchuan 750004, Chinayujq910315@163.com (J.-Q.Y.); 2 Key Laboratory of Protection, Development and Utilization of Medicinal Resources in Liupanshan Area, Ministry of Education, College of Pharmacy, Ningxia Medical University, Yinchuan 750004, China; 3 School of Food Science and Engineering, Ningxia University, Yinchuan 750021, China

**Keywords:** *Cistanche deserticola*, homology of medicine and food, intestinal moistening and laxation, intestinal motility, gut microbiota

## Abstract

*Cistanche deserticola* is a perennial parasitic herb of the Orobanchaceae family and a traditional Chinese medicinal material included in the homology-of-medicine-and-food category. It has traditionally been used to nourish the kidney and essence and to relieve constipation. Recent studies have investigated its potential effects on intestinal motility, gut microbiota, and fecal water regulation. This review summarizes the reported chemical constituents of *C. deserticola*, including phenylethanoid glycosides, polysaccharides, flavonoids, and sugar alcohols, and examines the available evidence regarding their potential contributions to laxative effects. Possible mechanisms involving intestinal motility, neurotransmitter-related signaling, gut microbiota and microbial metabolites, and aquaporin expression are discussed. Evidence is considered hierarchically: findings from identified *C. deserticola* preparations are distinguished from indirect findings involving other Cistanche species, isolated compounds, or multi-component herbal formulations, while proposed causal pathways are treated as hypotheses unless directly tested. The evidence base is predominantly preclinical, and human studies are limited; the specific contributions of individual constituents, the causal relationships underlying the proposed mechanisms, and the long-term safety of different preparations remain insufficiently established. This review also discusses challenges in raw-material standardization, evidence-based quality control, and functional food development, highlighting the need for further mechanistic, toxicological, and clinical studies to support the rational development of *C. deserticola*-based products.

## 1. Introduction

Constipation is a common gastrointestinal symptom characterized by hard or dry stools, infrequent bowel movements, straining, or a sense of incomplete evacuation. It may occur as a symptom of various conditions and should be distinguished from chronic constipation as a persistent clinical disorder. Constipation becomes more common with age, and its prevalence is also influenced by sex and other demographic factors [1,2]. Constipation can adversely affect health and quality of life and may coexist with conditions such as hemorrhoids and other systemic disorders; however, reported associations do not by themselves establish a direct causal relationship. Traditional Chinese medicine (TCM) is widely used in the management of constipation and related bowel symptoms, with Cistanche receiving attention for its potential laxative effects [3].

**Figure 1:**
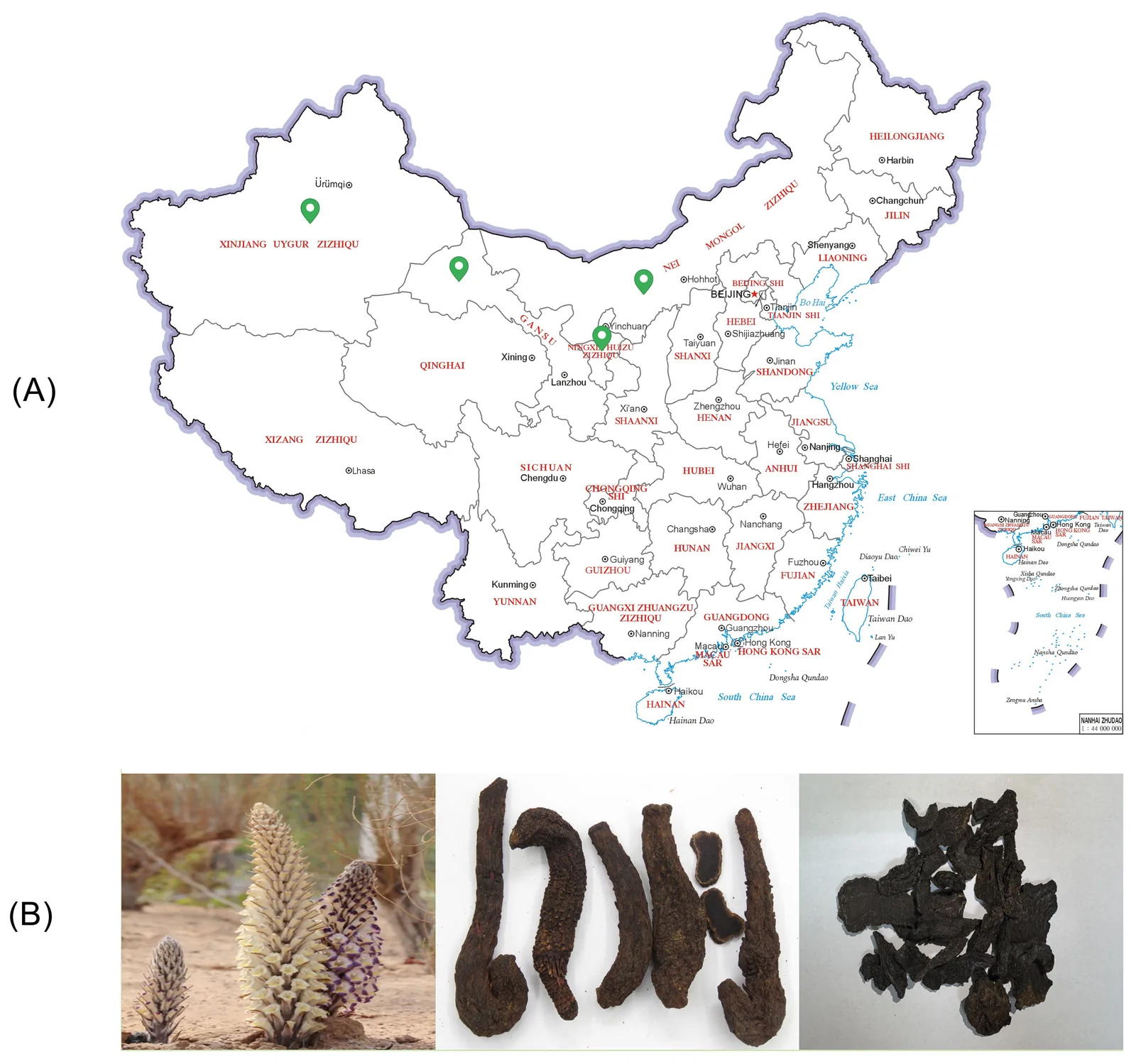
The distribution, morphology and decoction pieces of *Cistanche deserticola* ((**A**) source: the standard map service system of the Ministry of Natural Resources, http://bzdt.ch.mnr.gov.cn/index.html, map review number 6062445287; the base map only superimposed data, the boundary is not changed; (**B**) source: Chinese Plant Species Information System ‘iPlant’ http://www.iplant.cn/).

*Cistanche deserticola*, often known as desert ginseng or Rou Cong Rong in Chinese, is a member of the genus Cistanche in the family Orobanchaceae [4]. It is a perennial parasitic herb found in desert regions, including parts of China, North Africa, Mongolia, and the Middle East. Figure 1 shows the distribution, morphology, and decoction pieces of *Cistanche deserticola*. Reported activities include immunomodulation, antioxidant effects, gut microbiota modulation, and potential effects on bowel function [5]. Cistanche has a long history of use in Chinese medicine and was described in *Shen Nong’s Materia Medica*. It is used in traditional formulas for patterns associated with kidney deficiency and constipation. Its classification in China as a medicinal-and-edible substance has encouraged its use in both medicinal and food-related products; however, this designation should not be interpreted as evidence that every preparation is safe for all populations or under all conditions.

**Figure 2:**
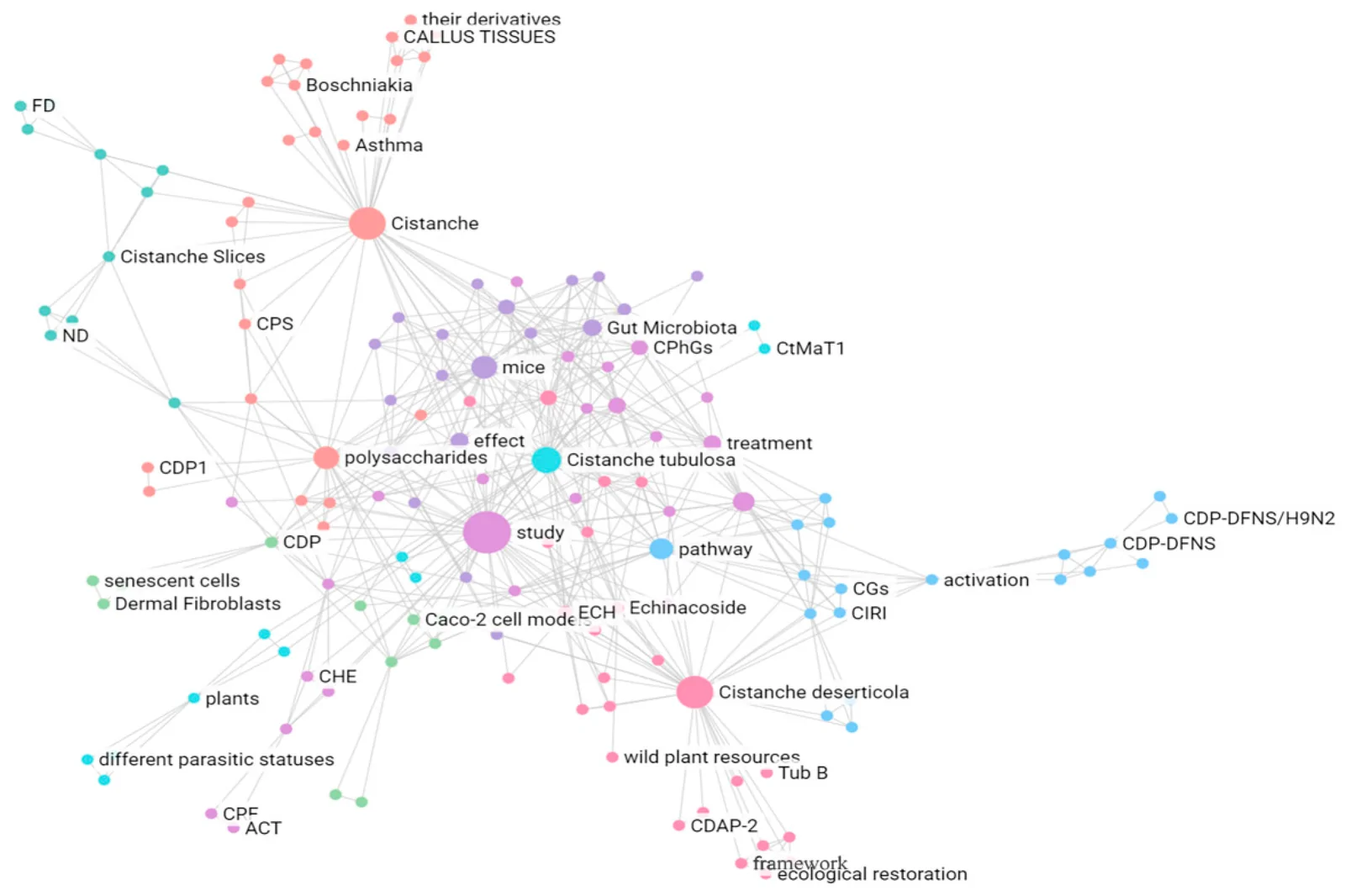
Cistanche research hotspot map.

Research on Cistanche has examined its chemical constituents, potential effects on bowel function, and applications in Chinese patent medicines, health foods, and functional foods. However, the strength of evidence varies across these areas, and findings from different species, isolated compounds, animal models, and multi-component formulations are not directly equivalent. Figure 2 shows research hotspots in the field of *Cistanche deserticola*. This review summarizes the reported constituents, laxation-related findings, proposed mechanisms, and product development, while distinguishing direct evidence for *C. deserticola* from indirect evidence and hypotheses.

For interpretation, evidence was considered in three categories: (1) direct evidence from studies testing identified *C. deserticola* preparations and reporting relevant outcomes; (2) indirect evidence from other Cistanche species, isolated constituents, or multi-component formulations, which may inform but cannot establish effects of *C. deserticola* itself; and (3) proposed mechanisms or causal links that have not been directly tested. Human findings were considered separately from preclinical evidence.

This narrative review used ResearchRabbit (https://www.researchrabbitapp.com/home; accessed on 15 March 2026) as the principal tool for literature discovery and citation-network exploration. The initial search used combinations of the following terms: (1) “*Cistanche deserticola*”, “Cistanche”, and “Rou Cong Rong”, and (2) “constipation”, “laxative”, “intestinal motility”, “fecal water content”, “gut microbiota”, “aquaporin”, “polysaccharide”, and “phenylethanoid glycoside”. Articles identified through the initial keyword searches were assessed for relevance and used as seed papers when they addressed Cistanche or a potentially relevant laxative-related outcome. ResearchRabbit’s “Similar Work”, “Earlier Work” (references), and “Later Work” (citing articles) functions were then used iteratively to identify additional publications connected to the seed papers. Newly identified records were screened by title and abstract, and potentially relevant articles were assessed using the available full text. Studies were considered for inclusion when they investigated *C. deserticola* or clearly identified Cistanche extracts/components and reported laxative-related outcomes or mechanisms in in vitro, animal, or human research. Duplicate records, reports without sufficient information to identify the tested material, and studies unrelated to laxative effects were excluded. Chinese-language publications and patents were considered where relevant, while human studies, animal experiments, in vitro studies, and patents were interpreted according to their different evidence types. Findings from other Cistanche species, isolated compounds not directly tested as *C. deserticola* preparations, and multi-component traditional formulations were identified as indirect evidence and were not treated as direct evidence for *C. deserticola*. Because ResearchRabbit expands searches through relationships among papers, this process was used for literature discovery and citation tracking rather than as a claim of exhaustive database-specific systematic searching.

For reproducibility, the principal seed papers identifiable from the references cited in this review were the reports on Cistanche-associated constituents and laxation-related outcomes, compound- and preparation-level mechanistic studies, the report on aquaporin expression in Cistanche-treated constipated rats, and the toxicological study of *C. deserticola* extract. The search was last updated in March 2026. Because the original narrative search did not maintain a separate prospective seed-paper register, this reference-numbered list is the seed set identifiable from the included manuscript rather than an exhaustive search log.

## 2. Traditional Chinese Medicine Practice of Cistanche for Intestinal Moistening

In traditional Chinese medicine (TCM), Cistanche is described as sweet, salty, and warm and is associated with the kidney and large-intestine meridians. Classical materia medica texts describe its traditional uses in patterns characterized by kidney yang deficiency, insufficiency of essence and blood, and constipation attributed to intestinal dryness [6]. For example, Ben Cao Hui Yan describes Cistanche as “warm but not hot” and as nourishing the life gate, strengthening kidney qi, and replenishing essence and blood [6]. The phrase “tonifying without harshness, moistening without purgation” is also used in traditional descriptions of this herb [7,8]. These statements summarize the traditional TCM understanding and historical indications of Cistanche; they should not be interpreted as clinical proof of efficacy or as evidence that all Cistanche species or preparations have equivalent effects [9].

Unlike the traditional descriptions of Cistanche, contemporary pharmacological studies have investigated specific constituents, including phenylethanoid glycosides, for possible effects on bowel function [10]. Some experimental reports describe changes in intestinal motility, fecal water content, intestinal mucus-related measures, or gut microbiota after treatment with Cistanche-associated materials or constituents [11,12]. However, these findings vary in the botanical source and type of material tested, and evidence from isolated compounds or other Cistanche species cannot by itself establish these effects for *Cistanche deserticola*. The proposed combination of intestinal moistening and tonifying should therefore be understood as a traditional therapeutic rationale, rather than a demonstrated synergistic pharmacological effect.

In TCM theory, constipation is commonly differentiated into patterns, including cold, wind, qi, heat, and deficiency patterns. Cold-pattern constipation is traditionally associated with internal cold and kidney yang deficiency, while treatment principles may include warming kidney yang and addressing intestinal dryness. Within this traditional framework, Cistanche is used for constipation patterns considered to involve kidney yang deficiency, insufficiency of blood and essence, or intestinal dryness [13]. These pattern descriptions and treatment principles reflect TCM theory and do not, on their own, establish efficacy under modern clinical criteria.

Traditional formulas may combine Cistanche with herbs such as Angelica sinensis, Cannabis seed, or Platycladus seed, with the intended purpose of addressing different aspects of a TCM pattern [14,15,16]. In traditional explanations, Angelica sinensis is associated with nourishing blood, Cannabis seed with moistening the intestines, and Cistanche with tonifying kidney yang and moistening dryness. Such descriptions explain the rationale for combining these herbs, but do not demonstrate pharmacological synergy or establish that the combination improves clinical outcomes beyond the effects of its individual components. The safety of a formula depends on its composition, dose, preparation, duration of use, and the characteristics of the person taking it; traditional use alone is insufficient to conclude that these combinations are low in toxicity or suitable for long-term use in older, postpartum, or chronically ill individuals. Cong Rong Run Chang Wan is a classical formula traditionally used for constipation associated with deficiency of essence and body fluids. Ji Chuan Jian, as recorded in *Jingyue’s Complete Works*, does not originally contain Cistanche, although Cistanche may be added in some later clinical practices. The composition and use of specific formulas should therefore be distinguished from evidence for *Cistanche deserticola* itself, and any claims of efficacy, synergy, or long-term safety require appropriate clinical and toxicological evidence.

## 3. Material Basis of Laxative Effect of *Cistanche deserticola*

Modern analytical techniques, including GC-MS and HPLC-Q-TOF-MS/MS [17], have identified diverse constituents in Cistanche, including phenylethanoid glycosides, iridoids, lignans, polysaccharides, amino acids and derivatives, flavonoids, and sugar alcohols. However, identification of a compound in Cistanche does not in itself establish laxative activity, and activity reported for an isolated compound or in another botanical context cannot automatically be attributed to *Cistanche deserticola*. Table 1 therefore summarizes selected compounds and laxation-related findings alongside the source and level of evidence, distinguishing direct evidence from indirect or background evidence [18,19,20].

### 3.1. Phenylethanoid Glycosides

Cistanche is known for its phenylethanoid glycosides (PhGs), which are typically made up of a phenylethanol aglycone connected to one or more sugar moieties [37]. The compound’s aromaticity, polarity, and reactivity are determined by its core structure, a phenyl ring with a two-carbon side chain (-CH_2_–CH_2_–OH). Rhamnose and glucose are the most common sugar moieties; arabinose and galactose are also occasionally found [38]. Additional substituents on the aglycone or sugars are examples of structural changes that improve antioxidant activity and affect neuroprotective, anti-inflammatory, and stress-response activities [39]. Certain PhGs increase structural variety by forming disaccharide or trisaccharide structures or by further esterifying to produce more complex glycosides.

### 3.2. Polysaccharides

With molecular weights ranging from tens of thousands to millions of Daltons, polysaccharides are linear or branching macromolecules made up of more than ten monosaccharides joined by glycosidic linkages [40]. Solubility, viscosity, stability, and bioactivity are determined by their monosaccharide composition, bond type, branching, and conformation.

Cistanche polysaccharides are mainly composed of glucose, fructose, galactose, arabinose, and rhamnose in varying ratios [41,42]. Studies of purified polysaccharides, including CDA-0.05, CCDP-1, and CCDP-2, have reported effects on small-intestinal motility, defecation time, stool number, and gut microbiota. These findings suggest possible laxation-related activity, but the extent to which they can be attributed to *C. deserticola* preparations depends on the source and characterization of the tested materials.

### 3.3. Flavonoids

Flavonoids are common plant polyphenols that have a 2-phenylchromone core and can be either free or glycosylated. Many subclasses (flavonols, flavanones, isoflavones, etc.) with various bioactivities are produced by variations in ring saturation, substituents, and glycosylation patterns [43].

Flavonoids such as quercetin, luteolin, vitexin, liquiritin, and naringin have been identified in Cistanche [44]. Some studies have suggested that these compounds may influence intestinal function; however, direct evidence demonstrating that flavonoids isolated specifically from *C. deserticola* exert laxative effects remains limited. Therefore, their possible contribution requires further verification.

### 3.4. Sugar Alcohols

Polyhydric substances with potent hydrophilicity and osmotic activity are known as sugar alcohols [45]. Compared with echinacoside, galactitol, a sugar alcohol reported in Cistanche, has been described as relatively abundant. Its osmotic properties provide a plausible explanation for effects on luminal water and stool hydration; however, the contribution of galactitol in specific *C. deserticola* preparations requires direct evidence.

### 3.5. Lignans and Iridoids

Cistanche also contains lignans and iridoids, which have been associated with antioxidant, antiviral, anti-inflammatory, and metabolic effects. However, direct laxative activity has not been established for these compounds, and their possible contribution to the overall effects of Cistanche remains speculative.

### 3.6. Other Considerations

Research on Cistanche increasingly considers multicomponent interactions in addition to isolated compounds. Plant-derived exosome-like vesicles and supramolecular assemblies formed during decoction have also been investigated [46,47]. These structures may affect component stability or interactions, but their roles in delivery, absorption, and coordinated pharmacological effects require further experimental validation.

## 4. Pharmacological Mechanisms of *Cistanche deserticola* for Laxative Activity

Pharmacological studies have reported changes in constipation-related parameters following administration of *Cistanche deserticola* preparations or materials associated with Cistanche, including changes in gastrointestinal motility, gut microbiota, and fecal-water regulation. However, the evidence comes from different types of test materials and does not support treating all reported mechanisms as established mechanisms of *C. deserticola* itself. Table 2 therefore separates findings from isolated compounds, combinations, multi-component mixtures, and specialized preparations, and indicates the limits of attributing these findings to the whole herb. The synthesis in Table 3 distinguishes direct evidence for the material actually tested, indirect evidence from isolated compounds or multi-component interventions, and hypothetical mechanisms that remain unverified. Animal experiments are classified as preclinical evidence, not clinical evidence. 

Mechanistic interpretations should be considered with caution because some findings derive from isolated compounds, combinations, multi-component mixtures, or specialized preparations rather than direct testing of a well-characterized *C. deserticola* preparation. Such findings may suggest possible pathways for further investigation, but they do not establish that the whole herb acts through the same mechanisms. Further studies using well-characterized *C. deserticola* preparations, with direct laxation-related endpoints and appropriate mechanistic validation, are needed.

### 4.1. Regulatory Effects of Cistanche deserticola on Intestinal Motility

Intestinal motility refers to the coordinated contractions and relaxations of intestinal smooth muscle. It includes peristalsis, segmentation, pendular movements, and defecation reflexes [48]. Pendular movements and segmentation contribute to mixing and absorptive contact, peristalsis propels intestinal contents, and the defecation reflex coordinates their final expulsion. Reduced intestinal motility is one pathophysiological factor associated with constipation, hard stools, and incomplete evacuation. Therefore, changes in intestinal motility may contribute to the laxative effects reported for Cistanche, although the extent to which Cistanche directly modulates these processes depends on the preparation and evidence available.

Intestinal motility is influenced by smooth muscle, neurotransmitters, hormones, and gut microbiota [50]. However, evidence for Cistanche-associated effects on these processes comes from specific formulations (Ji Chuan Jian, Qi Di Tongbian Fang, and Jichuanjia) or the isolated compound echinacoside, rather than direct mechanistic studies of a well-characterized *C. deserticola* preparation.

#### 4.1.1. Smooth Muscle Contraction

The smooth muscle of the intestinal wall is the direct executor of intestinal motility; its regular contraction and relaxation supply the mechanical force needed to move, mix, and evacuate intestinal contents. Both the intrinsic contractile machinery of muscle cells and the control of specialized interstitial cells of Cajal (ICC) in the gut are necessary for smooth muscle contraction [51]. Because intracellular Ca^2+^ fluctuations directly cause filament sliding and muscle contraction while regulating ICC electrical activity and neurotransmitter communication to smooth muscle, calcium ions are essential to this function [52].

Intestinal contractions are coordinated by interstitial cells of Cajal (ICCs), which are often described as gastrointestinal pacemaker cells. ICCs generate slow waves and contribute to the coordination of smooth-muscle electrical activity [53]. ICC dysfunction or abnormal distribution may be associated with irregular slow waves, reduced colonic transit, and defecation difficulties. Because ICCs are difficult to measure directly, c-kit mRNA is sometimes used as an ICC-associated marker, although it is not a direct count of ICCs. In a mouse study of the multi-herb formula Ji Chuan Jian, c-kit mRNA levels were significantly higher in the treatment group than in the model group [54]. This finding indicates a change in an ICC-associated marker after treatment with Ji Chuan Jian; it does not establish that *C. deserticola* alone increases ICC numbers or improves intestinal motility through this pathway, because the formula contains multiple herbs and the contribution of Cistanche was not isolated.

The calcium signaling pathway is a crucial regulator of smooth muscle contraction. A rapid increase in intracellular Ca^2+^ binds to the four sites of calmodulin, inducing conformational changes that activate myosin light chain kinase (MLCK) [59]. This catalytic activation phosphorylates the myosin light chain, triggering the ATPase activity of smooth muscle myosin, which hydrolyzes ATP to convert chemical energy into mechanical energy, allowing myosin to slide along actin filaments and achieve muscle contraction. In a study of mice treated with Qi Di Tongbian Fang, a multi-component formula, intracellular Ca^2+^ in colonic cells increased significantly; subsequent measurements also showed increases in calmodulin (CaM) and MLCK in colon tissue [55]. These findings support involvement of calcium-related signaling in the response to this formula, but they do not establish that *C. deserticola* alone produces these changes or that the Ca^2+^-CaM-MLCK pathway has been demonstrated for a specific *C. deserticola* preparation.

#### 4.1.2. Regulation by Neurotransmitters

Intestinal motility is regulated by a complex network of neurotransmitters, including excitatory mediators such as substance P (SP) and 5-hydroxytryptamine (5-HT), as well as inhibitory mediators such as nitric oxide (NO) and vasoactive intestinal peptide (VIP) [60]. However, the available evidence does not establish the complete sequence in which *C. deserticola* exposure stimulates enterochromaffin cells to release 5-HT, activates 5-HT4 receptors, promotes substance P release, and subsequently activates NK1 receptors and intracellular Ca^2+^ signaling. A study of echinacoside, an individual phenylethanoid glycoside associated with Cistanche, reported changes in SP and 5-HT4-related signaling markers together with a decrease in VIP in constipated mice [61]. These findings concern echinacoside and selected neurotransmitter-related endpoints; they do not demonstrate the entire proposed 5-HT-5-HT4-SP-NK1-Ca^2+^ cascade or establish this mechanism for a whole *C. deserticola* preparation. In addition, reported changes in 5-HT, SP, VIP, and NO in rats treated with Jichuanjia were obtained using a multi-component traditional Chinese medicine formulation rather than *C. deserticola* itself [56] and should therefore be regarded as indirect evidence. The broader proposal that Cistanche may influence these neurotransmitter pathways is supported mainly by mechanistic interpretation and review-level discussion [61]. Accordingly, general neurotransmitter regulation of intestinal motility is established physiology; changes in selected neurotransmitter-related markers have been reported for echinacoside and a multi-herb formula; and the specific sequence linking a *C. deserticola* preparation to 5-HT release, 5-HT4 activation, SP/NK1 signaling, and Ca^2+^-dependent contraction remains unverified.

### 4.2. Improvement of the Intestinal Microenvironment by Cistanche deserticola

Intestinal health is intimately related to the gut microbiota, which includes bacteria, fungi, viruses, and archaea. In order to maintain overall intestinal homeostasis, a balanced microbiota promotes digestion, nutritional absorption, intestinal barrier integrity, local immune control, and the production of metabolic products [62]. Microbial dysbiosis may be a contributing factor to constipation, since recent research shows that the abundance, composition, and diversity of gut microbiota varies significantly between patients with constipation and healthy controls [63]. Intestinal motility, hydration absorption, and fecal softness can all be negatively impacted by decreased probiotics, elevated pathogenic bacteria, and altered microbial metabolite profiles.

Cistanche polysaccharides may influence the gut microbiota by promoting the growth and activity of beneficial bacteria such as Lactobacillus and Bifidobacterium [64]. Polysaccharide fermentation by gut microorganisms can generate short-chain fatty acids (SCFAs), including acetate, propionate, and butyrate. SCFAs are extensively absorbed by the colonic epithelium and may influence electrolyte transport, epithelial energy metabolism, mucosal barrier function, intestinal motility, and G-protein-coupled receptor signaling [65]. Their effects on luminal water balance are therefore likely to involve multiple processes rather than a simple increase in osmotic pressure. Changes in SCFA production and utilization may alter epithelial and electrolyte transport and may contribute, together with changes in motility and barrier function, to the regulation of stool hydration and defecation. However, the extent to which these processes mediate the laxative effects of *C. deserticola* remains to be directly established.

Yali et al. reported that herbal dietary-fiber formulas for intestinal-moistening and laxation were associated with changes in gut microbiota and improvement in difficulty-in-defecation-related outcomes in mice [57]. This finding suggests that microbiota changes may accompany the effects of dietary-fiber-based interventions. However, because this study investigated a multi-ingredient herbal dietary-fiber formula rather than *C. deserticola* itself, it should not be regarded as direct evidence for a Cistanche-specific microbiota–SCFA mechanism. Studies of Cistanche polysaccharides and *C. deserticola*-derived preparations suggest that microbiota and microbial metabolites may participate in its intestinal effects, but the causal relationships among microbiota composition, SCFA metabolism, epithelial water/electrolyte transport, intestinal motility, and laxation remain to be clarified.

### 4.3. Regulation of Fecal Water Content by Cistanche deserticola

Constipation is associated with changes in stool hydration and intestinal transit. Sufficient luminal water helps keep feces soft and may facilitate evacuation. Some studies have examined aquaporin (AQP) expression in relation to Cistanche-associated effects on fecal water content; however, the available evidence does not establish that Cistanche directly regulates AQPs to increase fecal water content or produce laxative effects.

#### 4.3.1. Aquaporins

Aquaporins (AQPs) are a family of transmembrane water-channel proteins, with thirteen recognized subtypes in mammals [58]. Their expression throughout the digestive tract shapes fecal hydration and gastrointestinal function, and dysfunctional AQPs can result in dry, hard stools and constipation. In the colon, AQP3, located predominantly on the basolateral membrane of epithelial cells, governs fecal water content by mediating water exchange between the intestinal lumen and the blood vessels, whereas AQP4, which is also abundantly expressed in the colon, participates in intercellular water transport [66]. AQPs are passive water channels, and the direction and magnitude of net water movement depend on osmotic gradients, epithelial permeability, intestinal segment, and physiological conditions rather than AQP expression alone. Therefore, changes in AQP abundance do not necessarily indicate a corresponding change in net transepithelial water flux. Yu Hemei et al. reported decreased AQP3 and AQP8 expression in constipated rats treated with Cistanche extracts, suggesting that AQP-related pathways may be involved in the observed changes in fecal water content [67]. However, these expression changes alone do not establish that reduced AQP abundance directly attenuates colonic water reabsorption or determines the direction of water movement. The relationship between AQP expression and fecal hydration may also depend on the localization and functional status of individual AQP subtypes, as well as concurrent changes in intestinal motility, epithelial transport, and luminal osmotic conditions. Accordingly, AQP modulation should be regarded as a potential contributor to the laxative effects associated with Cistanche, rather than a confirmed mechanism of water retention or restoration of a physiological set point. Further studies measuring water transport and fecal hydration alongside AQP expression and localization are needed to clarify this relationship.

#### 4.3.2. Osmotic Regulation

**Figure 3:**
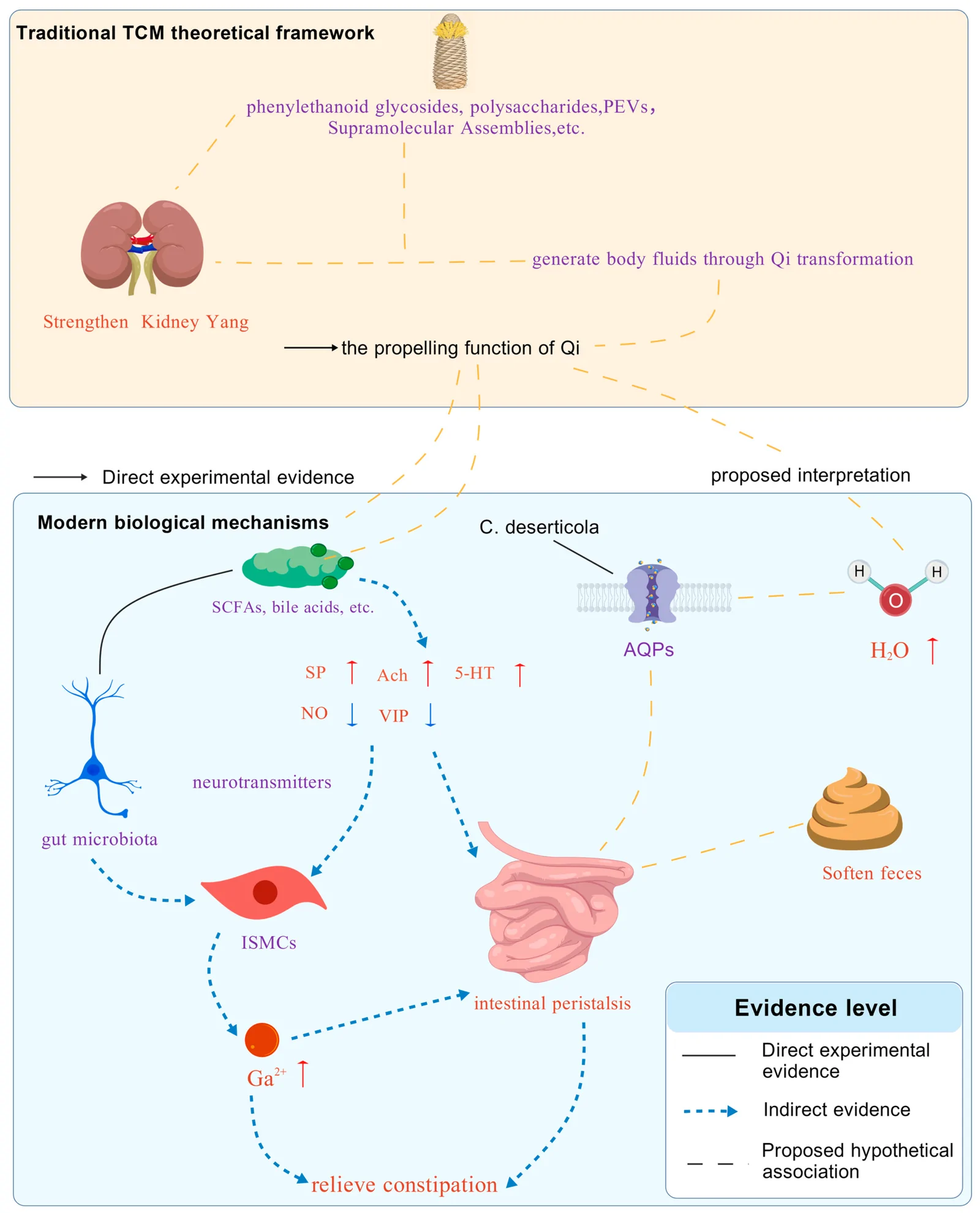
Mechanism of Cistanche in moistening the intestines and relieving constipation. Solid black arrows represent direct experimental evidence; dashed blue arrows represent indirect evidence; light yellow dashed arrows represent proposed hypothetical association. Upward arrows (↑) indicate upregulation or increased level, downward arrows (↓) indicate downregulation or decreased level.

Luminal osmotic conditions and fecal water content are related. An osmotic gradient can promote passive water movement into the intestinal lumen and may contribute to stool softening [68]. Conversely, fecal dryness can be associated with altered water and electrolyte transport, although these processes are not determined by osmotic pressure alone. Electrolytes, soluble substances, and microbial metabolites can influence luminal osmotic conditions. Cistanche-derived constituents may affect these processes, but the available evidence does not establish that Cistanche consistently increases luminal osmotic pressure or directly stimulates intestinal glands to release Na^+^, Cl^−^, and K^+^. These proposed mechanisms require further direct investigation [67]. The integrated regulatory relationships of these evidence-based mechanisms are summarized in Figure 3.

## 5. Safety

Safety findings should be interpreted separately by botanical species and tested material. The toxicological studies cited in relation to the inclusion of Cistanche in the medicinal-and-edible category concern C. tubulosa, including acute toxicity, genotoxicity, and 90-day repeated-dose testing; these results should not be taken as direct evidence for *C. deserticola*. For *C. deserticola*, Lu Wenting et al. reported toxicological testing of an extract, including mutagenicity-related assessments, acute oral toxicity, and 90-day repeated-dose toxicity in rats [69]. The available sources summarized here do not establish that the tested extracts, doses, and protocols were equivalent across the two species.

These species-specific preclinical findings provide preliminary information about the tested materials only. They do not establish the overall safety of all Cistanche products or support unrestricted long-term daily consumption. Further evaluation is needed regarding human exposure and tolerability, adverse events, dose range and duration, potential drug interactions, vulnerable populations, differences among extracts and preparations, and quality control, including standardization and possible contamination.

## 6. Product Development

**Figure 4:**
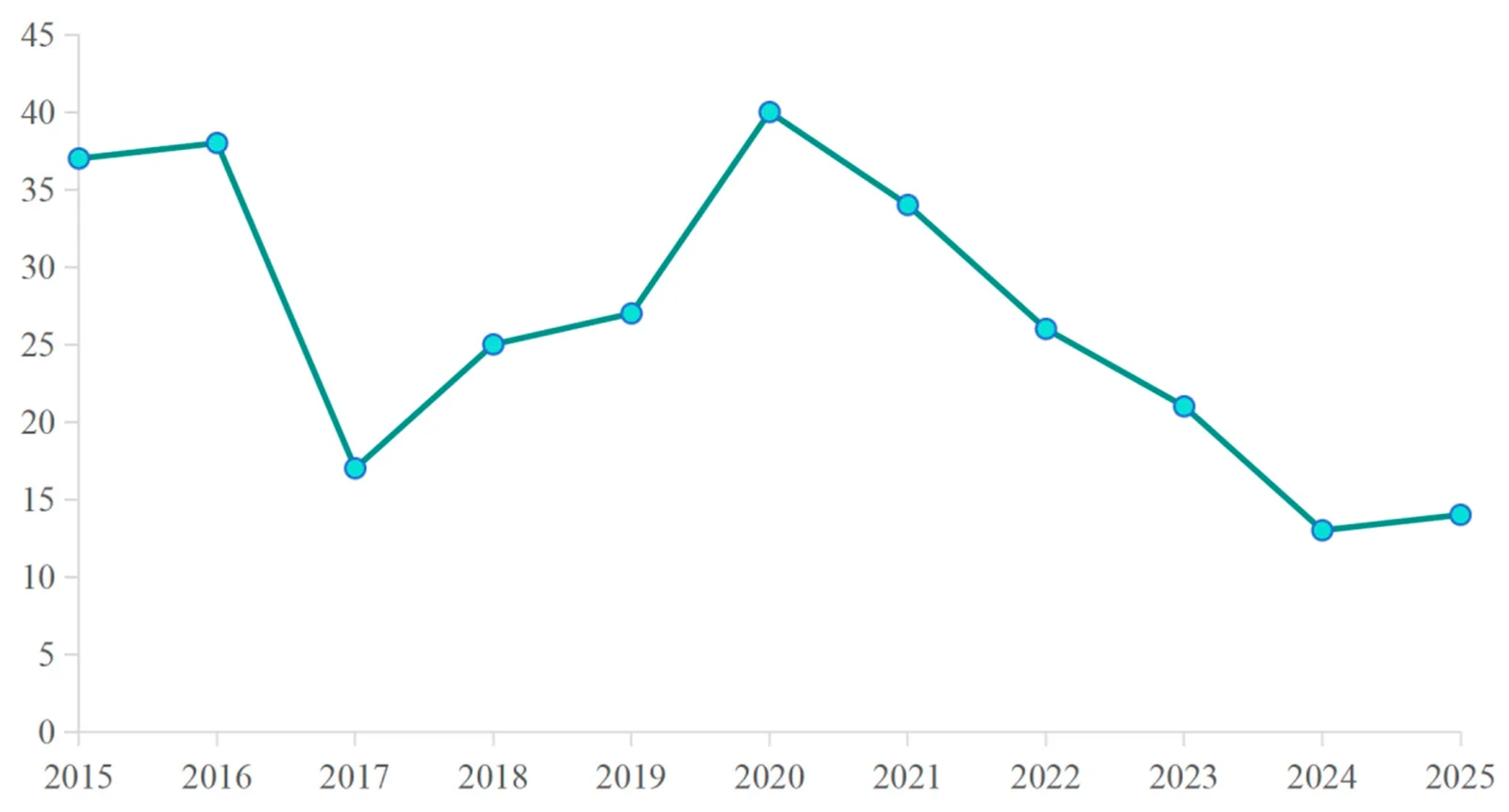
Patent applications of *Cistanche deserticola* from 2015 to 2025.

Cistanche has attracted interest as a potential ingredient in medicinal products and functional foods. Over the past ten years, 292 patents pertaining to Cistanche have been filed, nearly all of them in China, according to the Lens patent database. Figure 4 depicts the annual trend of *Cistanche deserticola* patent applications from 2015 to 2025.

According to the commercial and regulatory listings consulted in this review, Cistanche-containing Chinese patent medicines and health foods are available in multiple dosage forms, including capsules, tablets, injections, granules, oral liquids, and medicinal liquors. The databases cited in the original search listed 292 Chinese patent medicines and 72 approved health-food products containing Cistanche at the time of data collection. These are listing counts and may change over time; product registration or market availability does not itself demonstrate efficacy for constipation. The specific species, composition, and indication should be checked for each product [70,71,72].

Cistanche-containing products have been described in forms such as liquid drinks, tablet candies, and solid beverages. Table 4 summarizes selected examples and the product features reported in the cited sources. These examples document product formats and stated ingredients only; in the absence of product-specific controlled clinical evidence presented here, they should not be interpreted as proof of laxative efficacy, consumer acceptance, or suitability for routine use in people with constipation.

## 7. Limitations and Prospects

The evidence base is heterogeneous in botanical species, plant part and source, extraction and purification methods, chemical characterization, dose, treatment duration, experimental model, outcome measures, and study design. Many findings derive from isolated compounds, other Cistanche species, multi-herb formulas, or specialized preparations rather than a chemically characterized *C. deserticola* product. These differences limit direct comparison across studies and prevent simple pooling of outcomes. Most efficacy-related findings are preclinical, and the limited human evidence does not establish the efficacy, optimal dose, or long-term safety of *C. deserticola* for constipation.

Extrapolation to humans is particularly limited because animal models of induced constipation do not fully reproduce the causes, comorbidities, medication use, dietary patterns, or clinical endpoints of human constipation. Changes in biomarkers or proposed pathways do not necessarily demonstrate causal mediation of defecation outcomes. Future studies should use authenticated species, standardized and chemically characterized preparations, prespecified clinically relevant endpoints, appropriate controls, transparent reporting, and adequately designed human studies.

### 7.1. Raw Material Control

Cistanche typically grows in harsh environments with arid climate, low precipitation, long sunlight exposure, large diurnal temperature variation, such as sand dunes, dried riverbeds, and low-lying lake basins, particularly favoring mildly saline desert soils. With the development of Cistanche and related products, wild resources cannot meet human demand, leading to rapid expansion of artificial cultivation. However, cultivated Cistanche shows considerable variability in quality due to environmental factors, affecting consistency in laxative efficacy. Current pharmacopoeial standards use echinacoside and ferulic acid as quality control indicators, which still cannot fully reflect the real pharmacological contribution of the multi-component system, failing to meet the precise requirements of modern deep-processing products.

With Cistanche entering the list of medicinal and edible herbs, its application and processing are further promoted. To meet the huge demand for raw materials, standardized and systematic artificial cultivation is essential. First, systematic comparative studies of different species, hosts, origins, and ecological factors should be strengthened to construct optimal growth environments for different Cistanche species. Second, techniques such as LC-MS and component clustering analysis should be used to establish a comprehensive quality evaluation system for Cistanche. Finally, advanced technologies such as digital agriculture, precision irrigation, and host optimization should be applied to cultivation, harvesting, and processing to reduce quality differences between batches and improve overall quality controllability.

### 7.2. Material Basis Research

The laxation-related material basis of Cistanche remains incompletely defined. Studies have examined small- and large-molecule components, but differences in species, extraction methods, and experimental designs make it difficult to compare findings or identify constituents responsible for observed outcomes. Phenylethanoid glycosides have been investigated for possible laxation-related activity, but the available evidence does not establish them as universally confirmed key laxative components of *C. deserticola*. Interactions among constituents and possible additive or synergistic effects require direct testing.

Further investigation into TCM has revealed that the material foundation of efficacy may relate to the compounds’ surroundings in addition to small and large molecules. Consequently, exosome vesicles and self-assembled supramolecules have become emerging topics. Studies on Cistanche exosome vesicles and supramolecules are still in early stages. Techniques such as ultracentrifugation, ultrafiltration, infrared and ultraviolet spectroscopy can enhance understanding of Cistanche’s material basis for laxative effects. Moreover, multi-omics approaches, including metabolomics, proteomics, and single-cell sequencing, can systematically explore the material basis and mechanism, constructing a complete network of laxative mechanisms. This can reveal core active substances and provide a scientific basis for dosage form design.

### 7.3. Product Research and Development

Cistanche-containing products are available in several conventional dosage forms, including capsules, granules, and pills, as well as some beverage and candy formats. Whether one format improves adherence, palatability, bioavailability, or clinical outcomes over another requires product-specific evidence. The available information does not justify generalizing that existing products have slow onset or low bioavailability, or that newer formats are necessarily more convenient, safe, or effective.

Moreover, during product processing, loss of active components remains a significant issue. While quality control measures detect active component content, there is no standardization of loss from extract to final product. Active components are also prone to oxidation, and most products lack protection measures.

In extraction, modern techniques such as mechanochemistry, enzymatic extraction, and biomimetic methods should be applied to obtain controllable and directional extraction of active components, e.g., “enzyme + mechanochemistry” or “ultrasound/microwave + low-temperature extraction.”

Functional foods, such as cookies, drinks, fermented cheese, and nanoemulsions, are becoming more and more leisure-oriented and offer chances to update conventional administration techniques. By extending traditional decoction or infusion forms into more everyday-consumable formats, improvements in palatability, mobility, and methodical compound formulation creation can increase popularity and achieve deeper utilization of edible and medicinal resources.

Microencapsulation, nanoemulsions, solid lipid nanoparticles, solid dispersions, and other formulation techniques can protect delicate components, lessen oxidation and thermal degradation, enhance solubility and stability, and allow for controlled or colon-targeted release, increasing bioavailability and lowering batch variability. Clarifying appropriate populations and the best administration techniques is also essential. Subpopulations who respond best to Cistanche may be found through future research that combines clinical investigations with in vitro/in vivo gut motility evaluation, allowing for customized product strategies. Mechanism-based formulation design can support the scientific and individualized development of Cistanche functional foods by meeting various customer demands for daily consumption, precision nutrition, and long-term regulation.

## 8. Conclusions

As a medicinal-and-edible herb, Cistanche has traditionally been described as supporting intestinal moistening and relieving constipation, as well as tonifying essence and blood. Its medicinal use dates back to early Chinese medical texts. Cistanche contains phenylethanoid glycosides, polysaccharides, flavonoids, sugar alcohols, and other constituents. These compounds provide a basis for investigating its potential effects on bowel function; however, the contributions of individual constituents and the efficacy of different preparations require further evaluation before broad applications in functional foods and health products can be established.

Preclinical studies have reported changes in constipation-related outcomes following administration of certain Cistanche preparations or Cistanche-associated constituents, including outcomes related to intestinal motility, fecal water content, gut microbiota, and intestinal barrier function. However, the available evidence is heterogeneous and often derives from other Cistanche species, isolated compounds, or multi-component formulations. Therefore, these findings do not establish that *C. deserticola* itself directly regulates gut microbiota, intestinal motility, neurotransmitters, or gut hormones. These pathways remain potential mechanisms requiring confirmation in studies using well-characterized *C. deserticola* preparations. Other reported antioxidant, immunomodulatory, and metabolic effects also warrant further validation.

Important unresolved issues include variation in raw-material quality, the contribution of individual constituents, possible interactions among components, and changes in composition during processing. These questions require systematic investigation rather than assuming synergistic activity.

First of all, in terms of standardized control of raw materials, the current artificial planting of Cistanche is affected by the environment of the producing area, the host variety, and the harvesting and processing technology. The content of active components in different batches fluctuates greatly, and the current pharmacopoeia only uses a single index such as echinacoside to carry out quality control, which cannot reflect the synergistic effect of multiple components. Subsequently, based on liquid chromatography-mass spectrometry and multivariate chemometrics techniques, a comprehensive quality evaluation system including phenylethanoid glycosides, polysaccharides, sugar alcohols and flavonoids can be constructed to provide a stable raw material guarantee for large-scale food production.

Secondly, at the level of pharmacodynamic substances and mechanism of action, the existing research focuses on the independent pharmacological activity of monomer components and lacks systematic analysis of multi-component compatibility and synergistic intestinal moistening. The in vivo effects of emerging pharmacodynamic substances such as plant-derived vesicles and supramolecular assemblies are also still at an early stage. Therefore, future studies should clarify the relationships among Cistanche components, intestinal motility, aquaporin expression, gut microbiota, and microbial metabolites using integrated approaches such as metabolomics, proteomics, and single-cell sequencing. In particular, the proposed microbiota–SCFA axis should be tested as a potential mechanism rather than assumed to be a linear causal pathway, and evidence from *C. deserticola* should be distinguished from findings obtained with other Cistanche species, isolated compounds, polysaccharides from other sources, or multi-component traditional formulations. In addition, the in vivo delivery and intestinal targeting mechanisms of Cistanche extracellular vesicles and water-decoction supramolecular aggregates require further investigation to refine the modern pharmacological understanding of the multi-component, multi-target, and multi-pathway actions of Cistanche.

Product development may explore additional formats, including beverages, biscuits, chewing gum, and fermented foods. Such development should be guided by product-specific stability, quality, safety, dose, and efficacy data rather than by novelty of dosage form alone. Claims about targeted benefits for particular populations require supporting evidence.

The main limitations of the current literature are the predominance of preclinical studies, heterogeneity of tested species and preparations, inconsistent outcome measures, and limited clinical evidence. Findings from C. tubulosa, isolated compounds, multi-component formulas, or specialized vesicle preparations should not be generalized to *C. deserticola* without direct confirmation. Proposed mechanisms should be treated as hypotheses unless causal relationships have been tested, and market availability should not be equated with demonstrated clinical efficacy.

## Figures and Tables

**Table 1:** Reported constituents and laxation-related evidence associated with Cistanche species and selected compounds.

Phytochemical Type	Compound	Preparation	Model	Reported Laxation-Related Findings	Endpoint	Dosage	Evidence Status /References
Phenylethanoid glycoside	Acteoside	Purified from *Cistanche deserticola*; reference ionic-liquid aqueous two-phase separation method	Sprague–Dawley rats; pharmacokinetic and gut-microbiota studies	Alterations in gut microbiota composition and short-chain fatty-acid levels were observed; a direct laxative effect of this isolated compound is not established	Pharmacokinetics, gut-microbiota composition, short-chain fatty-acid levels	100 mg/kg orally (rat)	Indirect/associated evidence; direct laxative activity not established [21,22]
	Echinacoside	Ultrasonic-assisted ethanol extraction/isolated compound	Loperamide-induced constipated mice	Echinacoside can improve defecation parameters and intestinal function	Defecation indices, intestinal transit, colonic proteins and biochemical markers	Not available for single acteoside	Compound-level evidence; attribution to *C. deserticola* requires confirmation [23,24]
Sugar alcohol	Galactitol	Fraction isolated from Cistanche water extract by solvent extraction, chromatography and recrystallization	Diphenoxylate-induced constipated mice	Reported to shorten time to first defecation and increase fecal pellet number, fecal dry weight and small-intestinal propulsion in a dosage-dependent manner	Time to first red feces, fecal pellet number/dry weight, small-intestinal propulsion rate, fecal water content	Oral 1.1–11% solution	Laxation-related activity reported for an isolated fraction/compound; [25,26,27]
	D-mannitol	Pure compound, oral	Human bowel-preparation and constipation-related studies	D-mannitol has well-established osmotic laxative effects in clinical settings, yet there is no evidence confirming it as a constituent of *Cistanche deserticola*	Bowel-preparation score/adequate cleansing rate; time to first spontaneous defecation	10–20 g per dosage; 100 g for bowel preparation	Background evidence only; not direct evidence for *C. deserticola* [28,29,30,31,32]
Iridoid glycoside	Geniposide	Pure compound, oral administration	Charcoal-meal test and cecal-cannulation experiments	Oral geniposide induces diarrhea and enhances large intestinal propulsion, which does not verify geniposide as a laxative active constituent of *Cistanche deserticola*	Diarrhea incidence/onset; cathartic dosage; charcoal-meal propulsion and evacuation	dosage/ED50	Indirect evidence only; occurrence in *C. deserticola* and relevance to its effects require verification [33,34,35,36]

**Table 2:** Laxation-related findings reported for Cistanche-associated compounds and preparations.

Forms of *Cistanche*	Model	Dosage	Mechanism	Evidence Interpretation	References
Echinacoside	SPF male Kunming mice	30 and 70 mg/kg; 7 days	Expression of SP, 5-HT4-related signaling markers, AQP3, CFTR and PKA increased, whereas VIP expression decreased.	These findings describe laxation-related responses to echinacoside. They support a possible compound-level mechanism but do not independently establish the mechanism of a whole *Cistanche deserticola* preparation.	[48]
Acteoside and *C. deserticola* polysaccharide	SD male rats	Acteoside, 100 mg/kg; 21 days	Effects on intestinal microbiota and a reported interaction with *C. deserticola* polysaccharide were described.	The findings concern acteoside and its reported relationship with the polysaccharide; they should not be interpreted as direct evidence for a single-compound mechanism of the whole herb.	[49]
*C. deserticola* polysaccharide plus echinacoside	SD male rats	Polysaccharide, 400 mg/kg for 20 days; echinacoside, 100 mg/kg for 1 day	Reported to promote echinacoside absorption and affect intestinal microbiota.	These results relate to a combined intervention. The individual contributions of the polysaccharide and echinacoside cannot be separated from the reported findings.	[50]
Quercetin	SD male rats	10, 25 and 50 mg/kg; single dose	Changes in muscarinic acetylcholine receptor (mAChR)-related signaling were reported.	These are quercetin-level findings and do not establish that a well-characterized *C. deserticola* preparation produces the same effects.	[51,52]
	SD male rats	10, 20 and 40 mg/kg; single dose			
Sucrose, fructose, betaine and glucose mixture	SD male rats	60, 200 and 600 mg/kg; 32 days	Reported to reduce FC-induced metabolic disorders and increase CYP8B1, FGF15, TGR5 and FXR expression, with effects on bile-acid synthesis and enterohepatic circulation.	This is a mixture-level finding. It does not establish that any individual constituent or *C. deserticola* itself acts through this pathway.	[53]
Cistanche-derived exosome-like nanoparticles (CELNs)	Male C57BL/6J mice	25, 50 and 100 mg/kg	Reported effects on the microbiota–GABA axis, including enrichment of GABA-producing bacteria and changes involving GABAA receptor signaling; the effect was blocked by bicuculline.	These findings relate to a specialized nanoparticle preparation and should not be generalized to conventional *C. deserticola* extracts or attributed to a specific constituent.	[54]

**Table 3:** Evidence-level synthesis of reported laxation-related mechanisms associated with Cistanche and related materials.

Proposed Mechanism	Species/Source	Preparation or Material	Model	Endpoints Reported	Evidence Type	Key Limitations	References
Intestinal motility/neurotransmitter-related signaling	Cistanche-associated compound	Echinacoside	Constipated mice	Defecation-related indices; SP, 5-HT4-related markers, VIP, AQP3, CFTR and PKA-related markers	Direct evidence for the tested compound in an animal model; indirect for *C. deserticola* as a whole	Animal evidence, not clinical evidence.	[48]
Gut microbiota/microbial metabolites	*C. deserticola* polysaccharide and acteoside reported in combination	Acteoside plus *C. deserticola* polysaccharide	SD rats	Gut microbiota-related outcomes and reported interaction between materials	Direct for the tested combination; indirect for the independent effect of either component	Combined intervention prevents attribution to one component.	[49]
Absorption and microbiota-associated effects	*C. deserticola*	*C. deserticola* polysaccharide plus echinacoside	SD rats	Echinacoside absorption and intestinal microbiota-related endpoints	Direct for the combined intervention in animals; indirect for isolated mechanisms	Contributions of polysaccharide and echinacoside cannot be separated; no clinical efficacy established.	[50]
Muscarinic acetylcholine receptor-related signaling	Compound-level evidence; quercetin occurrence	Quercetin (isolated compound)	SD rats	mAChR-related signaling markers	Indirect evidence for *C. deserticola*; compound-level preclinical evidence	Does not establish that *C. deserticola* preparations contain an effective dose or act through the same pathway.	[51,52]
Bile-acid metabolism/FXR–TGR5-related signaling	Multi-component material associated with Cistanche	Sucrose, fructose, betaine and glucose mixture	SD rats; fructose-induced metabolic model	Metabolic endpoints and CYP8B1, FGF15, TGR5 and FXR expression	Indirect, mixture-level animal evidence	Not a single Cistanche constituent or a well-characterized whole-herb preparation; mechanism and laxation-specific causality remain uncertain.	[53]
Gut microbiota–GABA axis	Cistanche-derived material; species and composition depend on cited preparation	Cistanche-derived exosome-like nanoparticles (CELNs)	Male C57BL/6J mice	Microbiota changes, GABA-related endpoints and response to bicuculline	Direct for the specialized preparation in an animal model; indirect for conventional extracts; not clinical evidence	Specialized nanoparticle preparation cannot be generalized to decoctions or foods; pathway involvement does not establish human efficacy.	[54]
Intestinal smooth-muscle/Ca^2+^–CaM–MLCK signaling	Multi-herb formula containing Cistanche-associated ingredients; not *C. deserticola* alone	Qi Di Tongbian Fang	Mice	Intracellular Ca^2+^, CaM and MLCK in colon tissue	Indirect evidence from a multi-component formula	Cistanche-specific contribution is not isolated; animal findings do not establish clinical efficacy.	[55]
ICC-associated marker/motility	Multi-herb formula; Ji Chuan Jian	Ji Chuan Jian	Mice	Colonic c-kit mRNA	Indirect evidence from a multi-herb formula	c-kit mRNA is not a direct count of ICC; effect cannot be attributed to Cistanche alone.	[56]
Gut microbiota and stool-related outcomes	Multi-ingredient herbal dietary-fiber formula	Herbal dietary-fiber formula for intestinal moistening and laxation	Mice	Gut microbiota and difficulty-in-defecation-related outcomes	Indirect evidence from a mixed intervention	Not a *C. deserticola*-specific test; microbiota association does not prove mediation.	[57]
Aquaporin expression/fecal hydration	Cistanche extract; species	Cistanche extract	Constipated rats	AQP3 and AQP8 expression; fecal-water-related observations	Direct evidence for the tested extract in an animal mode	Expression alone does not establish water flux direction or causal laxative mechanism; not clinical evidence.	[58]

**Table 4:** Representative Cistanche-containing products and reported product features.

Product	Product Name	Major Constituents	Reported Product Features	References
Liquid Drink	Desert *Cistanche* plant beverage	*Cistanche* extract	Liquid beverage format; Cistanche extract listed as an ingredient	[73]
Tablet Candy	*Cistanche* tabletting candy	*Cistanche* powder	Tablet-candy format; Cistanche powder listed as an ingredient	[68]
Solid Beverage	*Cistanche* Runyi Granules	*Cistanche* concentrated solution	Granule/solid-beverage format; concentrated Cistanche solution listed as an ingredient	[67]

## Data Availability

No new data were created or analyzed in this study. Data sharing is not applicable to this article.
